# Spatial variations in the warming trend and the transition to more severe weather in midlatitudes

**DOI:** 10.1038/s41598-020-80701-7

**Published:** 2021-01-08

**Authors:** Francisco Estrada, Dukpa Kim, Pierre Perron

**Affiliations:** 1 Centro de Ciencias de la Atmósfera, Universidad Nacional Autónoma de México, Ciudad Universitaria, Circuito Exterior, 04510 Mexico, DF Mexico; 2 Institute for Environmental Studies, Vrije Universiteit, Amsterdam, The Netherlands; 3 Programa de Investigación en Cambio Climático, Universidad Nacional Autónoma de México, Mexico, Mexico; 4 Department of Economics, Korea University, 145 Anam-ro, Seongbuk-gu, Seoul, 02841 South Korea; 5 Department of Economics, Boston University, 270 Bay State Rd., Boston, MA 02215 USA

**Keywords:** Attribution, Atmospheric science, Climate change

## Abstract

Due to various feedback processes called Arctic amplification, the high-latitudes’ response to increases in radiative forcing is much larger than elsewhere in the world, with a warming more than twice the global average. Since the 1990’s, this rapid warming of the Arctic was accompanied by no-warming or cooling over midlatitudes in the Northern Hemisphere in winter (the hiatus). The decrease in the thermal contrast between Arctic and midlatitudes has been connected to extreme weather events in midlatitudes via, e.g., shifts in the jet stream towards the equator and increases in the probability of high-latitude atmospheric blocking. Here we present an observational attribution study showing the spatial structure of the response to changes in radiative forcing. The results also connect the hiatus with diminished contrast between temperatures over regions in the Arctic and midlatitudes. Recent changes in these regional warming trends are linked to international actions such as the Montreal Protocol, and illustrate how changes in radiative forcing can trigger unexpected responses from the climate system. The lesson for climate policy is that human intervention with the climate is already large enough that even if stabilization was attained, impacts from an adjusting climate are to be expected.

## Introduction

The attribution of climate warming to anthropogenic forcing was soundly established at the global and hemispheric scales through observation- and model-based methods^1–5^. At the subcontinental and finer regional scales, attribution studies rely mostly on climate models’ projections^6^. The attribution issue and description of the trends caused by changes in external forcing are relevant at these scales to understand the physical mechanisms of change, the consequences of anthropogenic climate change and the appropriate policies. An important recent research topic concerns the effects of rapid warming in the Arctic^7,8^ over midlatitudes’ weather and climate^9^, e.g., changes in the extremes of precipitation, hot-dry events and severe winters^8^. Research efforts focused on understanding the physical mechanisms and investigating whether these events are caused by natural variability or mark the onset of recurring features of the new climate driven by changes in regional climatic trends^10–12^. Attribution is of particular interest since such events can have considerable socioeconomic impacts and foster the attention of the public, media and decision-makers^13^.

Arctic amplification (AA) causes increases in external radiative forcing to produce faster increases in near-surface temperatures in Northern Hemisphere (NH) high-latitudes than elsewhere^7^. Although not completely understood, local and remote processes are known to be contributors, e.g., sea ice melting, reduction in albedo, clouds, downward longwave radiation, heat and moisture transport from tropical convection^14^. AA is present year-long but is seasonal being stronger in autumn and winter^15^. The rapid warming of the Arctic produced extensive and non-uniform losses in sea-ice coverage^16–18^. The AA intensity varied over time and since the 1990s it was accompanied by a lack of warming in NH-midlatitudes^9,19^. This pattern of warm Arctic cold continents/Eurasia (WACCE) was proposed as strong evidence that some mechanism offsets the warming in NH-midlatitudes^9,20^.

The warming and sea-ice loss in the Arctic can influence the weather and climate of near and remote regions^9–11^. The main dynamic pathways on NH-midlatitudes are changes in storm tracks, the jet stream and in planetary waves^11^. The first relates to how low-frequency extratropical variability is influenced by variability modes. In the NH-North Atlantic, those that can shift storm tracks equatorward when in a negative phase are the North Atlantic Oscillation (NAO) and the Arctic Oscillation (AO)^21^, causing severe winters in Eurasia and the US. The pattern is similar to that of WACCE^11,17,22^. Second, the temperature differences between Arctic and midlatitudes impact the polar jet stream. Decreases in temperature contrast cause large meanders in jet stream inducing polar air masses into midlatitudes and more persistent weather patterns^19^. Third, large expansions of ice-free sea areas in autumn and thinner sea ice in early winter produce increases in geopotential height thickness and reduced meridional gradient affecting the polar jet stream^8^, increasing the probability of blocking situations and the meridional transport of polar air masses^9^. Anomalous warmth in regions of the Arctic were linked to extremes in midlatitudes: those in the Barents-Kara Sea and in the East Siberian-Chukchi Sea associated to severe winters in East Asia and North America, respectively^9,16,17,23^. The AA can also influence midlatitudes summer extreme events via quasi-resonant amplification likely linked to AA, hence to anthropogenic warming^24,25^. Three robust links are: a decrease in the near-surface equator-to-pole gradient, a decline in late-spring to early-summer snow cover extent and reduced effect of tropical ENSO forcing on summer atmospheric circulation^10^. Still, large uncertainties remain about their impacts^13,26^.

Whether anthropogenic influences are factors driving climate/weather patterns in midlatitudes can be investigated by analyzing the spatial–temporal patterns of the response to increases in external forcings, the main common features of the regional temperature trends. Recent attribution studies showed that total and anthropogenic forcings are characterized by two marked changes in trend slope^3,4,27,28^, which are imparted to global and hemispheric temperatures, producing a temporal co-breaking pattern interpreted as a strong causality effect from anthropogenic forcing to temperatures^5^. The first is a marked increase in the rate of both forcings and warming near 1960, inducing the onset of the sustained global warming. The most recent change in slope is that produced by a slowdown in total radiative forcing since the mid-1990s, mainly as a consequence of the effects of the Montreal Protocol on CFCs and the reduction in methane emissions caused by changes in agricultural production in Asia^3,29,30^. This contributed to the so-called hiatus in global warming^7^, more precisely a period with a positive though reduced rate of warming^31,32^. Three broad groups of hypotheses to explain the hiatus have been put forward^33,34^, though as described later it is most likely caused by multiple factors: low-frequency natural variability; artifacts produced by deficiencies in temperature datasets and statistical methods; and changes and omissions in external radiative forcing. In the first group, the explanations relate to the warming trend being masked by the effect of low-frequency oscillations produced by coupled ocean–atmosphere processes and heat exchange between the ocean and the atmosphere^35–38^. Some of the main modes of variability that could have masked the warming trend since the 1990s are the Pacific Decadal Oscillation (PDO) and the Atlantic Multidecadal Oscillation (AMO), as well as North Atlantic Oscillation (NAO)^35,36,39,40^. The existence of the hiatus itself has been questioned with the argument that it is an artifact of biases in temperature records caused by measurement changes and errors, as well as the lack of coverage in the poles and Africa^41–43^. There is evidence supporting that neither natural variability nor data biases can fully account for the slowdown in the warming rate of global temperatures^28,31^. Another argument is that the slowdown could be due to the inadequate applications of statistical methods^44,45^. However, using formal univariate and multivariate structural change tests, when low-frequency oscillations are accounted for, the slowdown is a significant feature in global and hemispheric temperatures^4,28^. Evidence for the hiatus is also provided by recent climate models’ simulations^31^. Finally, a significant slowdown in the rate of growth in the total radiative forcing around 1990 has been documented^3,28^. The main causes were the effects of the Montreal Protocol over CFCs emissions^29^, a pause in methane emissions probably due to changes in agricultural production in Asia^46^, as well as increased loadings of atmospheric aerosols due to industrialization in Asia^47^; all of which are of anthropogenic origins. The effects of the Montreal Protocol and the pause in methane emissions alone account for 0.25 W/m^2^ (0.32 W/m^2^ if atmospheric aerosols are also included) in 2010. This reduction in radiative forcing is similar in magnitude to a full-amplitude solar cycle forcing and about 15% of the increase in total radiative forcing since 1880^3^. The importance of the Montreal Protocol to the slowdown observed and the future warming at the global and regional scales has been recognized, with a global cooling effect near 0.1 °C in 2013 and about 1.0 °C in 2050^3,48–51^. The Montreal Protocol has been hailed as the most effective international effort to date for reducing global warming^29,50^.

We contribute to this debate by extending current attribution studies to an explicit spatial context. Using recent methods to estimate common breaks in panel data with the stochastic component having a factor structure, we analyze the trends, common breaks and common factors of near-surface annual temperature series and produce a geographical world classification based on the characteristics of their estimated trends. Our results provide evidence about how anthropogenic forcing and feedback processes produced spatial changes in the magnitude and distribution of the warming in ways that are consistent with increasing extreme weather events in NH-midlatitudes.

## Data and methods

The temperature series used are version 2.0 of Cowtan and Way^42^, which applies kriging methods to impute missing data and offers a spatial resolution of 5° × 5° for the whole world (N = 2592 grid points). Temperature data records are sparse over some parts of the world such as the Arctic and Africa; how to account for the induced uncertainty and which are the optimal methods for infilling data in these regions are ongoing topics of investigation^42,43^. According to recent research, the magnitude of the warming in the Arctic region may be underestimated over the period 1998–2012^43^. As mentioned in the following sections, our analysis focuses on the general shape of regional warming trends and not on the precise estimated magnitude of the warming rate. Hence, while our estimates of the magnitude of warming rates may be influenced by this uncertainty, the conclusions presented here are robust to this problem. Temperature data are converted from monthly to annual by simple averaging. The sample period used is 1901 ~ 2016 (T = 116). To represent the most important sources of inter-annual global and hemispheric natural climate variability, we consider the following indices^52–56^: the AMO, the Southern Oscillation Index (SOI), the NAO, the PDO and the Arctic Oscillation (AO). We filter out the impacts of some modes of natural variability from each temperature series (see “Methods”). It is important to note that these natural variability indices are based on climate variables such as sea surface temperatures that are also influenced by natural and anthropogenic forcing. Although in some cases efforts are done to reduce the influence of external forcings (i.e., AMO is usually linearly detrended), part of this influence remains^35^. While separation of external forcing and natural variability in these indices is imperfect, it has been shown that they are useful in purging out low-frequency oscillations while retaining the trend produced by the response to changes in external forcing^3,27,28,37,39^. What is important is to get rid of the most of the low frequency movements associated with natural variability in order to get a more precise estimate of the break date. Natural forcings such as volcanic eruptions and solar irradiance have mostly transitory effects and, hence, no impact on our analysis, which is based on trends and can correct for short-term fluctuations.

Our statistical model is derived from empirical evidence^3,28,57^, which shows that global mean surface temperature is well characterized as a linear trend with two slope changes plus a stationary stochastic component. Thus, for the panel of filtered temperature series ${\text{y}}_{{{\text{it}}}}$, we suppose that each series consists of a deterministic trend, ${\text{d}}_{{{\text{it}}}}$, a linear function in time with two slope changes, and a stochastic component, ${\text{u}}_{{{\text{it}}}}$. The dates of slope changes (or break dates) are denoted by ${\text{T}}_{1} {\text{ and T}}_{2}$. They are assumed common across all locations. The stochastic component, ${\text{u}}_{{{\text{it}}}}$ is assumed to have a common factor structure. The common factors, a vector ${\text{f}}_{{\text{t}}}$, are stationary stochastic processes capturing co-movements in ${\text{u}}_{{{\text{it}}}}$. The factor loadings, a vector ${\uplambda }_{{\text{i}}}$, represent the impacts of common factors on each location. The part of ${\text{u}}_{{{\text{it}}}}$ not related to ${\text{f}}_{{\text{t}}}$ is an idiosyncratic error ${\text{e}}_{{{\text{it}}}}$. Thus,

1 $$ {\text{y}}_{{{\text{it}}}} = {\text{d}}_{{{\text{it}}}} + {\text{u}}_{{{\text{it}}}} , $$

$$ {\text{d}}_{{{\text{it}}}} = {\text{c}}_{{\text{i}}} + {\text{a}}_{{{\text{i}}0}} {\text{t}} + {\text{a}}_{{{\text{i}}1}} \left( {{\text{t}} - {\text{T}}_{1} } \right)^{ + } + {\text{a}}_{{{\text{i}}2}} \left( {{\text{t}} - {\text{T}}_{2} } \right)^{ + } , $$

$$ {\text{u}}_{{{\text{it}}}} = {\text{f}}_{{\text{t}}}^{^{\prime}} {\uplambda }_{{\text{i}}} + {\text{e}}_{{{\text{it}}}} , $$

where $\left( { } \right)^{ + }$ is the positive part of the argument. While the break dates are common across locations, the break coefficients, ${\text{a}}_{{{\text{i}}1}} {\text{ and a}}_{{{\text{i}}2}}$ are heterogeneous and locations can have a different warming pattern. To estimate the break dates, a panel method seems a priori preferable. However, Kim^58^ shows that this need not hold with strong cross-sectional dependence in the common factors. To obtain efficient estimates Kim^59^ advocates estimating the break dates jointly with the common factors. The segmented trend can be projected out and the common factors and loadings be estimated as principal components. The method searches for the pair of break dates that gives the smallest sum of squared residuals. With estimates of the break dates, the break coefficients are estimated via ordinary least squares. The common factors and factor loadings are the principal component estimates of the temperature series de-trended using the segmented trend.

To estimate the number of factors, we use the eigenvalue approach of Ahn and Horenstein^60^, valid for a panel of stationary time series. Hence, we can estimate the number of factors after projecting out the trend, but the estimates of the trend also depend on the number of factors through the estimation of the break dates. To circumvent this problem, we use the number of factors coherent with the break date estimates. We first estimate the break dates ignoring common factors. We then de-trend each series using these initial break date estimates and estimate the number of factors, which suggests one factor. We re-estimate the break dates with one common factor via Kim’s^59^ method, de-trend each series with the new break date estimates, and re-estimate the number of factors, which is again one. Hence, we conclude that there is one common factor (see “Methods” and the Supplementary Information for details).

## Results

The estimated common break dates for the panel of 2592 time series are 1954 and 1993. The 95% confidence intervals are tight, [1950, 1958] and [1991, 1995] (see “Methods”). These dates are in agreement with previous estimates^3–5^ based on global or hemispheric temperatures. While not all temperature series from all grid cells experienced a statistically significant break at those dates, it is reasonable to extrapolate and investigate the patterns of changes over grid cells using these global dates. This approach allows to quantify the differences in warming in the three segments across regions.

**Figure 1:**
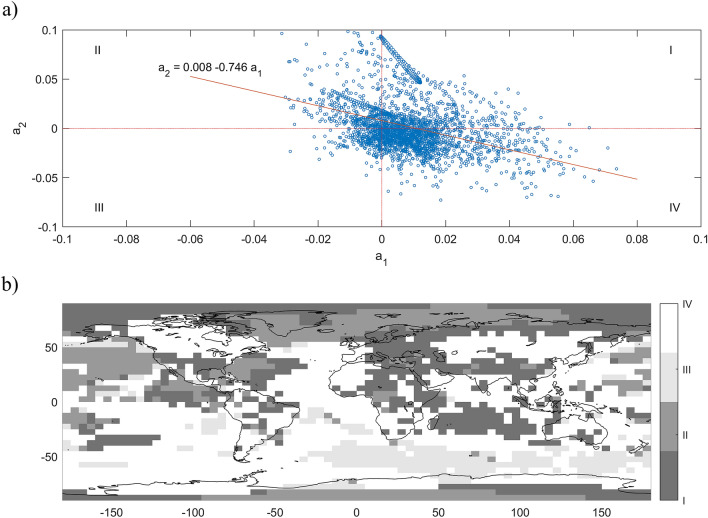
Spatial patterns of annual warming trends. (**a**) Shows a scatter plot of the estimates of ${\text{a}}_{{{\text{i}}1}} {\text{ and a}}_{{{\text{i}}2}}$ in Eq. (1), for the changes in slope parameters for the first (1954) and second break (1993), respectively. Depending on the signs of ${\text{a}}_{{{\text{i}}1}} {\text{ and a}}_{{{\text{i}}2}}$, there are four cases: I (+, +), II (−, +), III (−, −), and IV (+, −), each of which corresponds to a quadrant in the scatter plot. (**b**) Presents the geographical distribution of the four cases in a gray map, with case I being the darkest gray and case IV being white. This figure was created using MATLAB R2018a (https://www.mathworks.com/).

**Figure 2:**
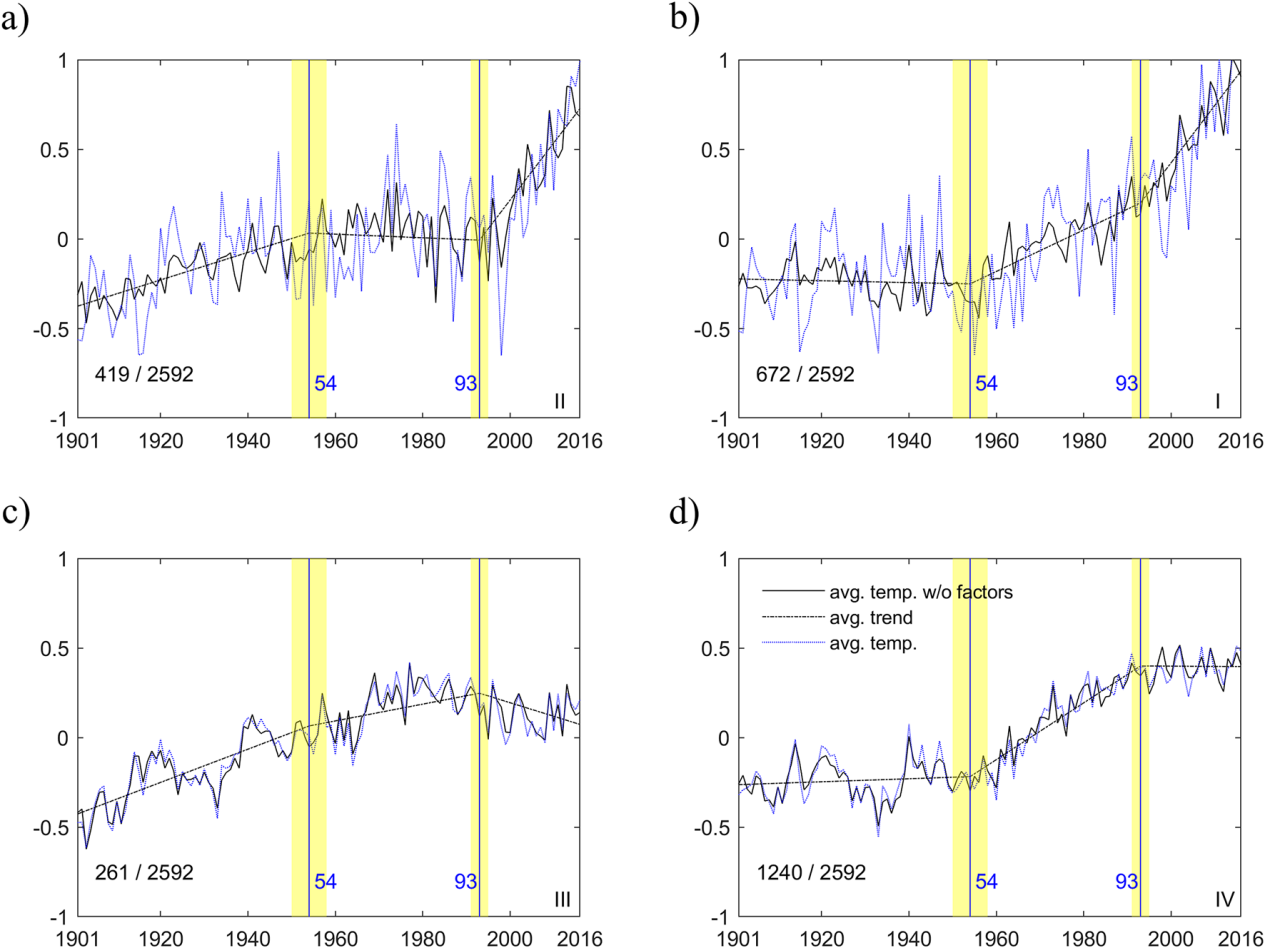
Average annual warming trends. The dash-dot line in each panel shows the average trend for each of cases I ~ IV in Fig. 1. (**a**–**d**) Denote the cases II, I, III and IV, respectively. The dotted line is the average of annual temperatures and the solid line is the average of annual temperatures without the common factor part (${\text{f}}_{{\text{t}}}^{^{\prime}} \lambda_{i}$). The two vertical lines indicate the estimated break dates with the shaded interval being the asymptotic 95% confidence intervals. The fraction in the bottom left corner of each panel shows the number of series for each case.

Based on these global break date estimates, the trend function ${\text{d}}_{{{\text{it}}}}$ is estimated from the temperature series of each grid cell. Of interest are the slope coefficients, ${\text{a}}_{{{\text{i}}1}}$ and ${\text{a}}_{{{\text{i}}2}}$, i.e., the changes in warming trend. Figure 1a shows the scatter plot of the estimates of ${\text{a}}_{{{\text{i}}1}}$ and ${\text{a}}_{{{\text{i}}2}}$ along with a fitted regression line. The slope coefficients are negatively correlated. A large (small) change in the post first break slope is on average followed by a small (large) change after the second break. This result suggests changes in the pattern of warming across regions and time. To keep things manageable yet informative about regional patterns, we group the grids according to the signs of the estimates of ${\text{a}}_{{{\text{i}}1}}$ and ${\text{a}}_{{{\text{i}}2}}$, with four cases: I (+, +), II (−, +), III (−, −), and IV (+, −), each corresponding to a quadrant in the scatter plot. Table 1 reports the values of the average slopes for the three segments and four cases. Figure 1b shows the geographical distribution of the four cases (case I being the darkest gray through case IV being white). Figure 2 plots the average trend for each quadrant. Also reported is the proportion of each cases. Case IV (Fig. 2d) is the most common, showing a small rise until 1954, followed by a large increase from 1954 until 1993 and then a very slight decrease. This is quite similar to the pattern for global temperatures, though the post-1993 hiatus is not as severe, a slowdown in warming instead of a pause.

**Table 1:** Average trend slopes.

	I		II		III		IV	
	Slope	$\% \Delta$	Slope	$\% \Delta$	Slope	$\% \Delta$	Slope	$\% \Delta$
1901 ~ 1954	− 0.0005		0.0077		0.0093		0.0008	
1955 ~ 1993	0.0116	2328	− 0.0010	− 113	0.0047	− 49	0.0158	1784
1994 ~ 2016	0.0320	176	0.0319	3242	− 0.0076	− 261	− 0.0001	− 100

A broken linear trend ${\mathbf{d}}_{{{\mathbf{it}}}} = {\mathbf{c}}_{{\mathbf{i}}} + {\mathbf{a}}_{{{\mathbf{i}}0}} {\mathbf{t}} + {\mathbf{a}}_{{{\mathbf{i}}1}} \left( {{\mathbf{t}} - {\mathbf{T}}_{1} } \right)^{ + } + {\mathbf{a}}_{{{\mathbf{i}}2}} \left( {{\mathbf{t}} - {\mathbf{T}}_{2} } \right)^{ + }$ with ${\mathbf{T}}_{1} = 1954$ and ${\mathbf{T}}_{2} = 1993$ is fitted for each temperature series. The average of those trends are computed for each quadrant in Fig. 1a, labelled I, II, III, and IV. The slope for 1901–1954 is ${\mathbf{a}}_{{{\mathbf{i}}0}}$, ${\mathbf{a}}_{{{\mathbf{i}}0}} + {\mathbf{a}}_{{{\mathbf{i}}1}}$ for 1955–1993, and ${\mathbf{a}}_{{{\mathbf{i}}0}} + {\mathbf{a}}_{{{\mathbf{i}}1}} + {\mathbf{a}}_{{{\mathbf{i}}2}}$ for 1994–2016. The values of the slope are reported in columns labelled “slope”. The percent changes in the slope are reported in columns labelled “$\% {{\varvec{\Delta}}}$”.

Figures 1, 2 summarize the spatial–temporal patterns of warming since the early twentieth century. About 74% of the grid cells (cases I and IV, shown in Fig. 2b,d, respectively) experienced more rapid warming after the break in 1954, covering most land and sea surface and about half the Arctic region. About 10% of the grid cells (case III, Fig. 2c) warmed at a slower pace, which could be linked to the absorption of heat from the southern oceans where this pattern occurs more frequently. Slight cooling occurred for grids cells in quadrant II (16%, Fig. 2a), mainly covering the northeast Pacific Ocean, some part of the north Atlantic Ocean and about half of the Arctic Ocean including the Kara-Barents and Greenland seas and Baffin Bay.

After the second break in 1993, 58% of the world’s grid cells experienced a slowdown in warming (cases III and IV, shown in Fig. 2c,d, respectively). These correspond to most of the Atlantic and southern oceans and a considerable fraction of continental land, including large parts of the NH midlatitudes. Note that for grids in quadrant II, which mainly cover the Oceans, the pattern is one of slight cooling in 1954–1993 followed by the most rapid increase across all cases (Fig. 2a). This is consistent with the hypothesis that much of the Oceans absorbed a large amount of warming during the rapid global increase. Some tipping point occurred in 1993, after which these regions, especially the Artic, experienced a much more rapid warming of ~ 3.2 °C per century (case II, Table 1), in agreement with the period of rapid warming caused by AA reported in the literature^8,9,19^. The largest changes in warming occurred in areas covering the Kara-Barents and Greenland seas and the Baffin Bay which had previously experienced slight cooling; hence a 3,242% increase in the warming rate (Table 1, case II). Most other Arctic regions had an increase of ~ 176% in the warming rate (Table 1, case I), including the Chukchi and East Siberian seas. The warming and sea ice loss in the Barents-Kara sea has been linked to persistent cold spells and to a much higher probability of severe winters in Eurasia^12,17,61^, while warming in the Chukchi, East Siberian and Greenland seas has been linked to cold winters in North America and Europe^62–64^. Due to sparse data in the Arctic region, recent literature suggested that the magnitude of the warming rates for this region could be underestimated^43^. Note that all the Arctic region in our analysis falls in cases I and II, for which the post 1993 warming rate shows an increase. The effect of underestimating the warming trends due to spare data in the Arctic could affect the reported magnitudes but not the classification of this region as case I and II, which is robust to this problem. At the same time, parts of the midlatitude areas in North America and Eurasia experienced a hiatus period contemporaneous with the decrease in total radiative forcing (case IV). As discussed above, physical mechanisms have been proposed that connect the warming pattern of warm Arctic and cold continents with the occurrence of extreme weather during winter and summer in midlatitudes^9,10^. The spatial pattern created by the second break in the common warming trend bares a considerable resemblance with negative AO patterns associated with widespread cold temperatures in NH^8,9,11,65^. The analysis of winter temperatures (Supplementary Figs. S1–S2) shows the same general spatial–temporal patterns but with even larger diverging trends between polar and midlatitude regions.

**Figure 3:**
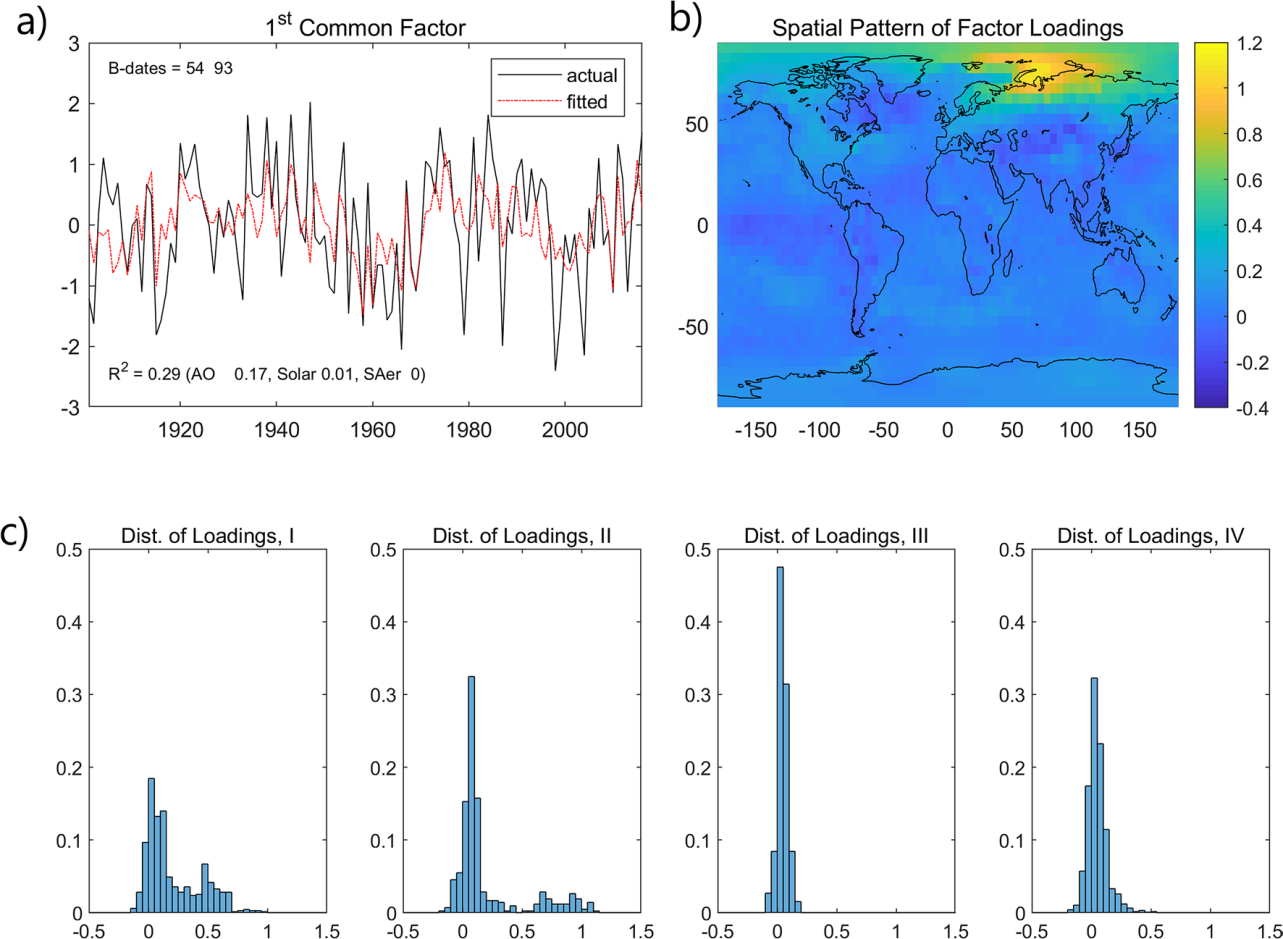
Time series and spatial pattern of the common factor with annual temperatures. The solid black line in (**a**) plots the common factor and the red dash-dot line is the fitted line from a regression of the common factor on a constant, AO and forcing factors (see “Methods”). The ${\text{R}}^{2}$ is 0.29 and the numbers in parenthesis are the marginal ${\text{R}}^{2}$ values of the three most significant regressors. (**b**) Displays the values of the factor loadings in a color map. (**c**) Shows the distribution of the loading values for each of the four cases, I ~ IV in Fig. 1. This figure was created using MATLAB R2018a (https://www.mathworks.com/).

Note that the model jointly estimates a stochastic component that captures co-movements in the noise component. Figure 2 (solid line) shows the temperature averages for each quadrant without the estimated common factor (${\text{f}}_{{\text{t}}}^{^{\prime}} \lambda_{i}$); the dotted line is the original average. These are noticeably different in cases I and II, while much closer in III and IV. Hence, the common factor impacts more regions with a positive warming trend post-1993 (I and II) than those with a hiatus (III and IV). We analyze the estimated common factor (Fig. 3a) to assess its relation to variability modes or radiative forcing components. The red dash-dot line, plotted along with the common factor, represents the fitted value from a regression using a constant, AO and forcing factors (see “Methods”). The main contribution comes from AO and the ${\text{R}}^{2}$ is 0.29, but the fitted line mostly follows the low frequency movements of the common factor. The values of the factor loadings are displayed in a color map (Fig. 3b). The common factor loads mostly in the Arctic with the maximum values occurring near the Barents-Kara sea. Figure 3c shows the distribution of the loading values for each cases. The loadings are near zero for III and IV while they are spread and right-skewed for I and II, with some values above one, indicating where the common factor has larger influence.

Our analysis separates two types of spatial–temporal patterns, reflecting climate (i.e., long term trends) and weather components, which influence regional temperatures worldwide and are related to mechanisms proposed to connect polar warming to midlatitudes weather and climate. The first is determined by the spatial effect of anthropogenic warming and manifest itself as a low-frequency movement causing the meridional temperature gradient to decrease since the 1990s, a pattern similar to that of WACCE. The second represents the co-movement in the stochastic component related to natural variability and modulates regional warming trends. Figure 3a,b show that AO is closely related to the estimated common factor; both share similar low-frequency movements, characterized by a negative oscillation phase starting in the 1940s, changing to positive in the 1960s, followed to negative in the 1990s and positive since the 2000s. The spatial pattern of the common factor is also broadly similar to that of AO, with positive maxima over the Siberia/Barents-Kara sea and North America (smaller magnitude) and a minimum over Greenland^66^. When in a negative phase, the AO pattern has been linked to severe winters in Eurasia and North America. These similarities between the common factor and AO are also present when analyzing regional winter temperatures (Supplementary Fig. S3). Hence, a climatological component, which remained unidentified^9,19^ and we here associate with the pause in warming caused by a slowdown in anthropogenic forcing, can make these events more likely. The interplay of these two spatial–temporal patterns can influence weather in the midlatitudes via mechanisms proposed in the literature and when in phase they can lead to extreme weather conditions.

## Conclusions

The spatial–temporal changes documented show four main types of warming patterns, with the presence/absence of the recent hiatus. The spatial patterns determined by the presence/absence of the slowdown in the warming can explain the WACCE pattern and the divergence in the warming trends between midlatitudes and the Arctic, features previously ascribed to some “unaccounted mechanism”^9^. We show that they are due to the spatial patterns produced by the warming slowdown along with AA. The hiatus is absent mostly in the Arctic region, which has been warming due to increases in external radiative forcing, AA feedback processes, which more than offset the decrease caused by the reduced rate of increase in total radiative forcing. The divergent trends produced by AA in the Arctic and the hiatus in parts of the midlatitudes contribute to a larger reduction of the thermal contrast between these regions. These changes could lead to severe weather in midlatitudes through physical mechanisms; e.g., changes in storm tracks. The effects of these diverging warming trends are modulated by a natural variability common factor similar to AO.

The reduced growth rate of total radiative forcing since the early 1990s is partly the result of successful international mitigation actions (e.g., the Montreal Protocol). The spatial–temporal evolution of warming shows that while temperature trends over large parts of the world can decrease as a result, others are subject to strong feedback processes (e.g., AA) and will keep warming. What this study conveys is that, while globally, there was a hiatus induced by reduced globally aggregated external forcings, at the regional scale there are clear differences in temperature changes not directly linked to such forcings but rather indirectly via transmission through regions; e.g., increased rapid warming in the Artic associated with a less rapid increase in the rest of the Northern hemisphere. Hence, our observation-based analysis indicates a clear spatial and temporal inter-dependence in the global warming. The divergent warming trends can have profound impacts on the weather of near and remote locations. Changes in anthropogenic forcing can have unexpected impacts on climate and weather even if international mitigation efforts are successful, underlying the need of adaptation and risk-reduction strategies at the local and regional levels.

## Methods

### Data

The temperature series, version 2.0 of Cowtan and Way^42^, were obtained from https://www-users.york.ac.uk/kdc3/papers/coverage2013/index.html. This is a version of the HadCRUT4 dataset with data gaps infilled by optimal interpolation methods (kriging).

The data sources for the most important natural sources of inter-annual global and hemispheric climate variability are: AMO, http://www.esrl.noaa.gov/psd/data/timeseries/AMO/, SOI, https://crudata.uea.ac.uk/cru/data/soi/soi_3dp.dat, NAO, https://www.esrl.noaa.gov/psd/gcos_wgsp/Timeseries/NAO/, PDO, https://www.ncdc.noaa.gov/teleconnections/pdo/, AO, https://climexp.knmi.nl/data/iao_slp_ext.dat.

All series are normalized such that their Euclidean norm equals $\sqrt T$. The radiative forcing are available from the NASA Goddard Institute for Space Studies^67^ (https://data.giss.nasa.gov/modelforce/).

### Filtering

Since our concern is with low-frequency movements, we filter out the impacts of the main long-term natural modes of variability, AMO and PDO, from each temperature series. The results are similar when NAO and SOI are also filtered. Denote the temperature of location *i* at time *t* by ${\text{Temp}}_{it}$ and the filtered temperature series by ${\text{y}}_{it}$. The filtered series are obtained as the residuals from an Ordinary Least-Squares (OLS) regression of ${\text{Temp}}_{it}$ on a constant, AMO, and PDO, i.e., ${\text{Temp}}_{{{\text{it}}}} = {\hat{\text{c}}}_{{{\text{i}}0}} + {\hat{\text{c}}}_{{{\text{i}}1}} {\text{AMO}}_{{\text{t}}} + {\hat{\text{c}}}_{{{\text{i}}2}} {\text{PDO}}_{{\text{t}}} + {\text{y}}_{{{\text{it}}}}$, where ${\hat{\text{c}}}_{i0} , {\hat{\text{c}}}_{{{\text{i}}1}}$, and ${\hat{\text{c}}}_{{{\text{i}}2}}$ are the OLS estimates. The filtered temperature series ${\text{y}}_{{{\text{it}}}} = {\text{Temp}}_{{{\text{it}}}} - \left( {{\hat{\text{c}}}_{{{\text{i}}0}} + {\hat{\text{c}}}_{{{\text{i}}1}} {\text{AMO}}_{{\text{t}}} + {\hat{\text{c}}}_{{{\text{i}}2}} {\text{PDO}}_{{\text{t}}} } \right)$.

### Radiative forcing

The radiative forcing series cover the period 1850–2012, which we extend to the period 2013–2016 using an autoregressive model^4^. The forcing factors used as regressors to generate the fitted value in Fig. 3a are: the well-mixed greenhouse gases (carbon dioxide (CO_2_), methane (NH_4_), nitrous oxide (N_2_O), and CFCs), ozone (O_3_), solar irradiance, land use change, snow albedo, orbital change, direct effect of tropospheric aerosols, indirect effect of tropospheric aerosols, and stratospheric aerosols (SAer). Those that have an important effect are solar and SAer.

For a detailed description of the statistical model and methods please see the accompanying Supplementary Information document.

## Supplementary Information

Supplementary Information.

## Data Availability

The data that support the findings of this study are available from the corresponding author upon reasonable request.
