# Supplementary material for: Spatial variations in the warming trend and the transition to more severe weather in midlatitudes
Source: Sci Rep. 2021 Jan 8;11:145. doi: 10.1038/s41598-020-80701-7 (PMC7794460; doi:10.1038/s41598-020-80701-7)
Supplement: Supplementary file 1 — Supplementary Information. [file 41598_2020_80701_MOESM1_ESM.docx]

**Spatial variations in the warming trend and the transition to more severe weather in midlatitudes.**

**Francisco Estrada^1,2,3*^, Dukpa Kim^4^ & Pierre Perron^5^**

*^1^Centro de Ciencias de la Atmósfera, Universidad Nacional Autónoma de México, Ciudad Universitaria, Circuito Exterior, 04510 Mexico, DF, Mexico.*

*^2^Institute for Environmental Studies, Vrije Universiteit, Amsterdam, Netherlands.*

*^3^Programa de Investigación en Cambio Climático, Universidad Nacional Autónoma de México, México.*

*^4^Department of Economics, Korea University, 145 Anam-ro, Seongbuk-gu, Seoul, 02841 Korea.*

*^5^Department of Economics, Boston University, 270 Bay State Rd., Boston, MA, 02215, USA.*

**Corresponding author,* [*feporrua@atmosfera.unam.mx*](mailto:feporrua@atmosfera.unam.mx)

**Supplementary Information**

**Supplementary Information.**

**S1. Model**. First, let $w_{t}(T_{1},T_{2})=(1,t,\left( t-T_{1} \right)^{+},\left( t-T_{2} \right)^{+})'$ and $\Pi_{i}=\left( c_{i},a_{i0},a_{i1},a_{i2} \right)^{'}.$ Then, equation (1) can be written as $y_{\mathrm{it}}=w_{t}\left( T_{1},T_{2} \right)'\Pi_{i}+f_{t}^{'}\lambda_{i}+e_{\mathrm{it}}$, which in vector form is, $y_{i}=w\left( T_{1},T_{2} \right)'\Pi_{i}+F\lambda_{i}+e_{i}$, where $y_{i}=\left( y_{i1},\ldots,y_{\mathrm{iT}} \right)^{'}$, $F=(f_{1},\ldots,f_{T})'$, $W\left( T_{1},T_{2} \right)=\left( w_{1}\left( T_{1},T_{2} \right),\ldots,w_{T}\left( T_{1},T_{2} \right) \right)^{'}$, and $e_{i}=\left( e_{i1},\ldots,e_{\mathrm{iT}} \right)^{'}$. In matrix form $Y=W\left( T_{1},T_{2} \right)^{'}\Pi+F\Lambda+e$, where $Y=(y_{1},\ldots,y_{N})$, $\Pi=(\Pi_{1},\ldots,\Pi_{N})$, $\Lambda=(\lambda_{1},\ldots,\lambda_{N})$, and $e=(e_{1},\ldots,e_{N})$. Note that $T_{1}$ and $T_{2}$ denote generic break dates, while the true break dates are denoted by $T_{1}^{0}$ and $T_{2}^{0}$. Similarly, $\Pi$, $F$, and $\Lambda$ denote generic trend coefficients, common factors, and factor loadings, while the true ones are denoted with superscript 0.

**S2. Estimation of the break dates.** We use the break date estimator of Kim^1^, obtained as the solution of the following minimization problem,

$\min_{T_{1},T_{2},\Pi,F,\Lambda} trace[(Y-W\left( T_{1},T_{2} \right)^{'}\Pi-F\Lambda)'(Y-W\left( T_{1},T_{2} \right)^{'}\Pi-F\Lambda)]$.

Using his global method to solve the above problem. The minimum of the objective function for a given pair $T_{1}$ and $T_{2}$ is found by first concentrating out $\Pi$ and subsequently minimizing with respect to $F$ and $\Lambda$ via a principal component analysis. Thus, we search for pairs $T_{1}$ and $T_{2}$ that achieve the global minimum of the objective function.

**S3. Estimation of the break coefficients, common factors, and factor loadings.** Upon obtaining the break date estimates, say $\hat{T}_{1}$ and $\hat{T}_{2}$, we estimate the trend function via ordinary least squares: $\hat{\Pi}=\left( W\left( \hat{T}_{1},\hat{T}_{2} \right)'W\left( \hat{T}_{1},\hat{T}_{2} \right) \right)^{-1}W\left( \hat{T}_{1},\hat{T}_{2} \right)'Y$.

The third and fourth rows of $\Pi^{0}$ contain the break coefficients $a_{1}^{0}=(a_{11}^{0},\ldots,a_{N1}^{0})$ and $a_{2}^{0}=(a_{12}^{0},\ldots,a_{N2}^{0})$, respectively. Thus, the corresponding rows of $\hat{\Pi}$ are used to create Table 1, Figures 1 and 2. We estimate the common factors and factor loadings via a principal component analysis after removing the estimated trend functions from the temperature series. More specifically, our estimates for the common factors, $\hat{F}$, are the eigenvectors of $\hat{U}\hat{U}'/(NT)$ corresponding to the r largest eigenvalues, where $\hat{U}=Y-W\left( \hat{T}_{1},\hat{T}_{2} \right)\hat{\Pi}$. We normalize $\hat{F}$ such that $\hat{F}'\hat{F}/T=I_{r}$. The estimates for the factor loadings are simply $\hat{\Lambda}=\hat{F}'\hat{U}/T$. These estimates are used to construct Figures 2 and 3.

**S4. Confidence intervals.** Kim^1^ uses various assumptions to derive the asymptotic distribution of the estimates of the break dates, some of which are worth mentioning. Both common factors and idiosyncratic errors are stationary linear processes allowed to be auto-correlated but without an autoregressive unit root. The common factors and idiosyncratic errors are independent, and the idiosyncratic errors are mutually independent. Although not all series are required to have a break, the cross sectional average of the squares of break coefficients should converge to a strictly positive number as the size of the cross-section grows. The factor loadings should be of the same order of magnitude as the break coefficients.

The asymptotic distribution of the break date estimator, reported in Kim^1^, is given by

$T^{\frac{1}{2}}N^{\frac{1}{2}}(\hat{T}_{j}-T_{j}^{0})\underset{\to}{d}N\left( 0,\frac{4\breve{S}_{\mathrm{jj}}}{\left( 1-\tau_{j}^{0} \right)\tau_{j}^{0}\breve{A}_{\mathrm{jj}}^{2}} \right)$ for j=1 and 2,

where $\tau_{j}^{0}=T_{j}^{0}/T$, $\breve{S}_{\mathrm{jj}}=\lim_{N} \frac{1}{N}a_{j}^{0}M_{\Lambda}\Sigma_{e}M_{\Lambda}a_{j}^{0}'$, $\breve{A}_{\mathrm{jj}}=\lim_{N} \frac{1}{N}a^{0}{}_{j}M_{\Lambda}a_{j}^{0}'$, ${M_{\Lambda}=I}_{N}-{\Lambda^{0}}^{'}\left( \Lambda^{0}{\Lambda^{0}}^{'} \right)^{-1}\Lambda^{0}$, $a_{j}^{0}=(a_{1j}^{0},\ldots,a_{\mathrm{Nj}}^{0})$, $\Sigma_{e}=diag(s_{1}^{2},\ldots,s_{N}^{2})$, and $s_{i}^{2}$ is the long-run variance, i.e., $2\pi$ times the spectral density at zero frequency of $e_{\mathrm{it}}$ for $1\leq i\leq N$.

We compute the confidence intervals reported in the text by replacing the true parameters in $\tau_{j}^{0}$, $\breve{S}_{\mathrm{jj}}$, and $\breve{A}_{\mathrm{jj}}$ with their estimates. The long-run variance of the idiosyncratic error is estimated by a kernel based non-parametric method.

**S5. Number of factors.** Let U is an $T\times N$ panel of data assumed to have a factor structure such that $U=F\Lambda+e$, where $F$ and $\Lambda$ are $T\times r$ and $r\times N$, respectively. Let $\mu_{k}(U)$ denote the k^th^ largest eigenvalue of $\mathrm{UU}^{'}/(NT)$. Define the ratio of two adjacent eigenvalues: $\mathrm{ER}\left( U,k \right)=\mu_{k}(U)/\mu_{k+1}(U)$. Ahn and Horenstein^2^ propose an estimate of $r$, the number of factors, by $\hat{r}(U)=\underset{1\leq k\leq k_{m}}{\mathrm{argmax}} ER(U,k)$, and show the consistency of their estimator for some $k_{m}>r$.

Since Ahn and Horenstein’s method^2^ cannot be applied to trending series, we estimate the number of factors by the following iterative procedure. First, we estimate the break dates ignoring common factors, that is, we solve

$\min_{T_{1},T_{2},\Pi} trace[(Y-W\left( T_{1},T_{2} \right)^{'}\Pi)'(Y-W\left( T_{1},T_{2} \right)^{'}\Pi)]$.

Let $\tilde{T}_{1}$ and $\tilde{T}_{2}$ be these initial break date estimates. We then estimate the number of factors after de-trending each series using these initial break date estimates:

$\hat{r}(\tilde{U})=\underset{1\leq k\leq k_{m}}{\mathrm{argmax}} ER(\tilde{U},k)$ with $\tilde{U}=Y-W\left( \tilde{T}_{1},\tilde{T}_{2} \right)\left( W\left( \tilde{T}_{1},\tilde{T}_{2} \right)'W\left( \tilde{T}_{1},\tilde{T}_{2} \right) \right)^{-1}W\left( \tilde{T}_{1},\tilde{T}_{2} \right)'$Y, which turns out to be one in all our applications. Next, we re-estimate the break dates using Kim’s method^1^ with one common factor. Let $\hat{T}_{1}$ and $\hat{T}_{2}$ be the resulting break date estimates. We de-trend each series using the break dates $\hat{T}_{1}$ and $\hat{T}_{2}$, and re-estimate the number of factors, via:

$\hat{r}(\hat{U})=\underset{1\leq k\leq k_{m}}{\mathrm{argmax}} ER(\hat{U},k)$ with $\hat{U}=Y-W\left( \hat{T}_{1},\hat{T}_{2} \right)\left( W\left( \hat{T}_{1},\hat{T}_{2} \right)'W\left( \hat{T}_{1},\hat{T}_{2} \right) \right)^{-1}W\left( \hat{T}_{1},\hat{T}_{2} \right)'Y$, which turns out to be one again in all our applications. Therefore, we conclude that there is one common factor.

**S6. Results for winter temperatures.**

When converting the data from monthly to annual, the average is taken across winter months only (December from the previous year, January, and February). Once the panel of winter temperatures is obtained, the analysis is carried out exactly as in the main text. The results are qualitatively the same, except that the first break date is estimated at 1936. The results are reported in figures S1-S3.


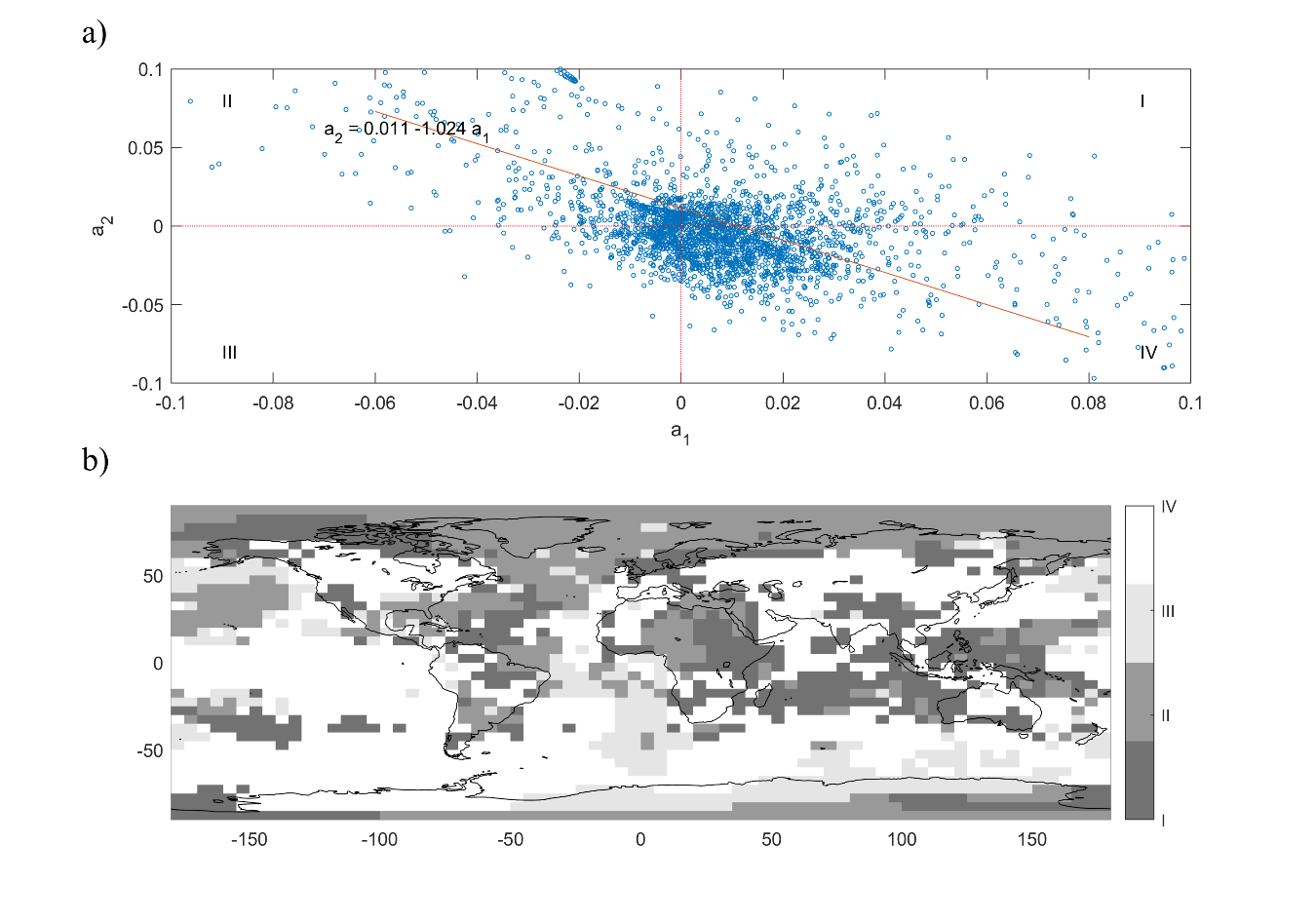


**Figure S1. Spatial patterns of winter warming trends.** Panel a) shows a scatter plot of the estimates of $a_{i1}\mathrm{and}a_{i2}$ in equation (1), for the changes in slope parameters for the first (1936) and second break (1993), respectively. Depending on the signs of $a_{i1}\mathrm{and}a_{i2}$, there are four cases: I (+,+), II (-,+), III (-,-), and IV (+,-), each of which corresponds to a quadrant in the scatter plot. Panel b) presents the geographical distribution of the four cases in a gray map, with case I being the darkest gray and case IV being white. This figure was created using MATLAB R2018a (https://www.mathworks.com/).


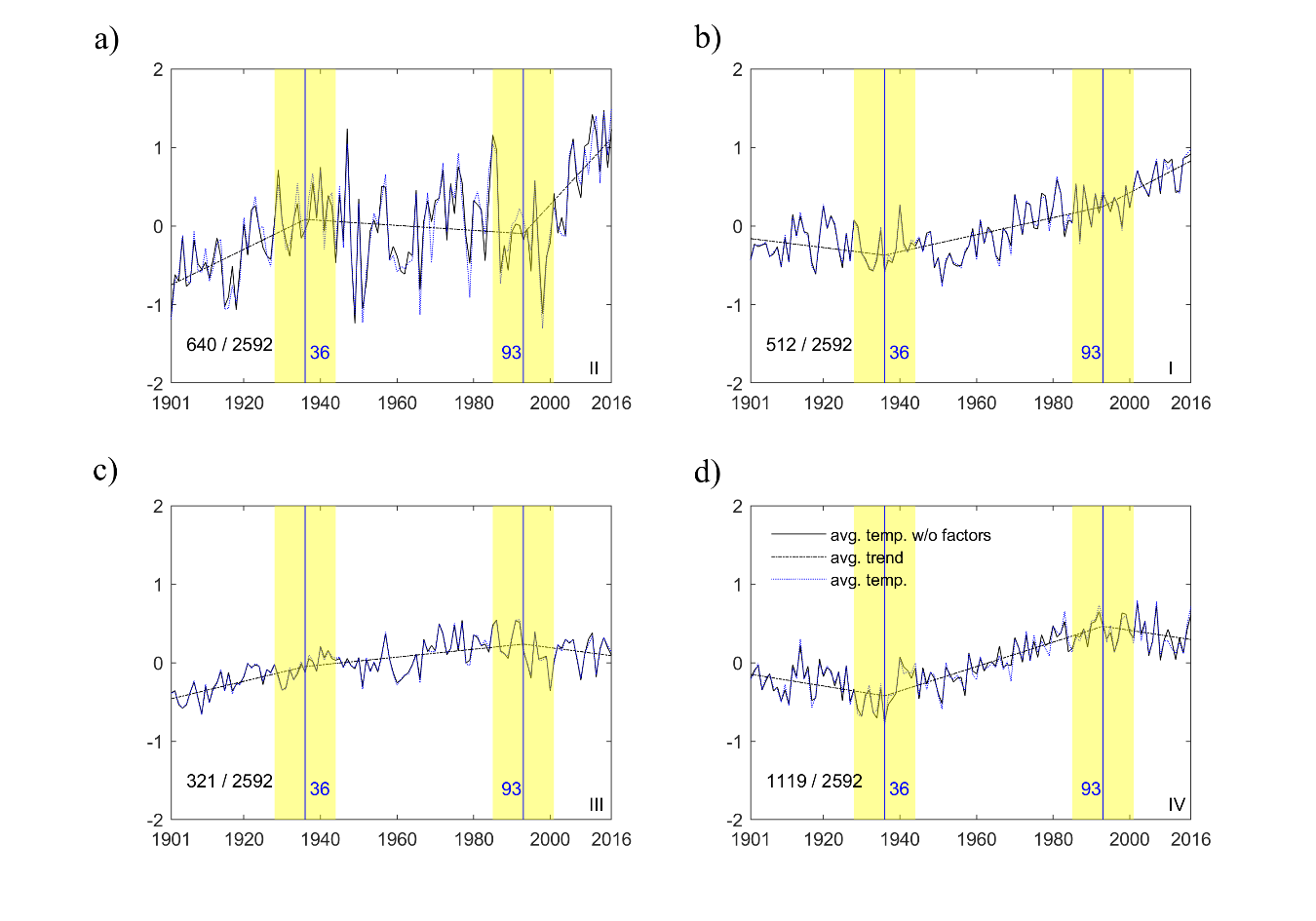


**Figure S2. Average winter warming trends.** The dash-dot line in each panel shows the average trend for each of cases I ~ IV in Figure 1. Panels a), b), c) and d) denote the cases II, I, III and IV, respectively. The dotted line is the average of winter temperatures and the solid line is the average of winter temperatures without the common factor part ($f_{t}^{'}\lambda_{i}$). The two vertical lines indicate the estimated break dates with the shaded interval being the asymptotic 95% confidence intervals. The fraction in the bottom left corner of each panel shows the number of series for each case.


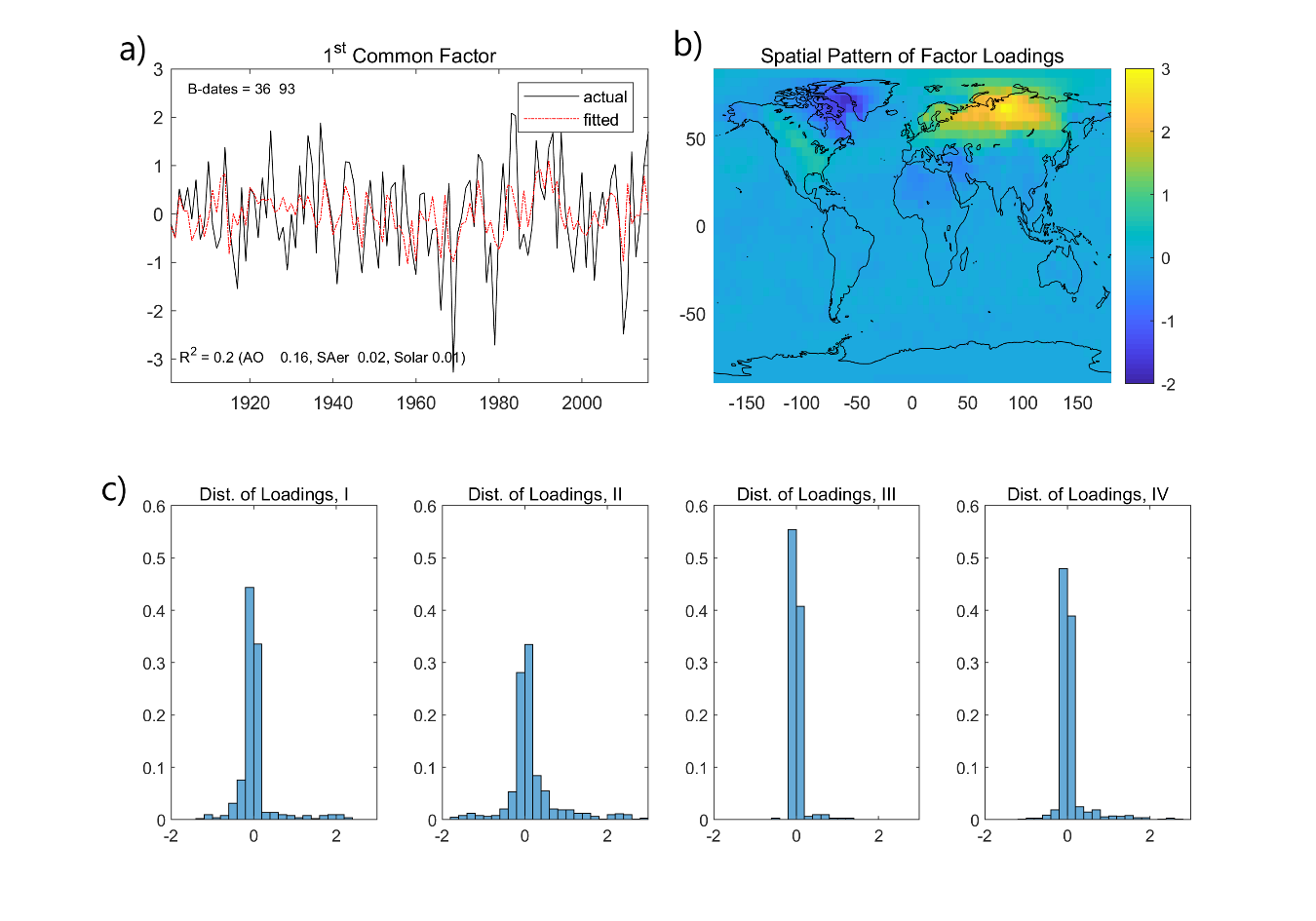


**Figure S3. Time series and spatial pattern of the common factor with winter temperatures.** The solid black line in panel a) plots the common factor and the red dash-dot line is the fitted line from a regression of the common factor on a constant, AO and forcing factors (see methods). The $R^{2}$ is 0.2 and the numbers in parenthesis are the marginal $R^{2}$ values of the three most significant regressors. Panel b) displays the values of the factor loadings in a color map. Panel c) shows the distribution of the loading values for each of the four cases, I ~ IV in Figure S1. This figure was created using MATLAB R2018a (https://www.mathworks.com/).

**S7. Results excluding polar data.**

A close look at Figure 1a reveals two sets of grids cells clustered in straight lines (top-left of quadrant I and bottom right of quadrant II extending to the lower part of quadrant I). The grid cells associated with these two lines correspond to data near the Arctic and Antarctic poles. Because data are sparse in these regions, the method of kriging creates great resemblance among temperature series of adjacent grid cells, which in turn makes the estimates of the trend parameters form almost straight lines in the scatter plot. Hence, we repeated all results without polar data, that is excluding data in latitudes above 85 or below -85. The break dates remain at 1954 and 1993 and all results are qualitatively similar; see Figures S4-S6.


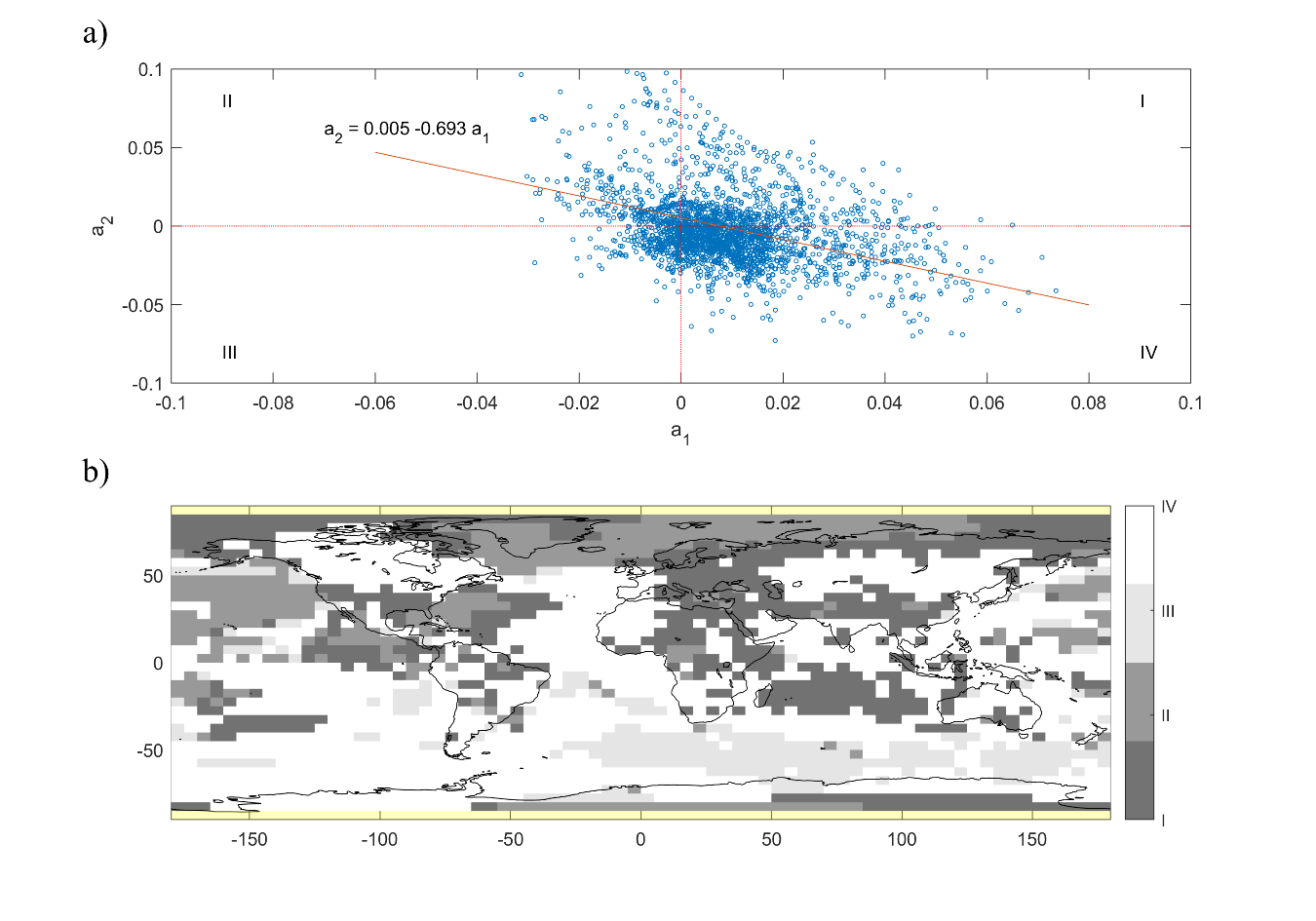


**Figure S4. Spatial patterns of annual warming trends excluding polar data.** Panel a) shows a scatter plot of the estimates of $a_{i1}\mathrm{and}a_{i2}$ in equation (1), for the changes in slope parameters for the first (1954) and second break (1993), respectively. Depending on the signs of $a_{i1}\mathrm{and}a_{i2}$, there are four cases: I (+,+), II (-,+), III (-,-), and IV (+,-), each of which corresponds to a quadrant in the scatter plot. Panel b) presents the geographical distribution of the four cases in a gray map, with case I being the darkest gray and case IV being white. This figure was created using MATLAB R2018a (https://www.mathworks.com/).


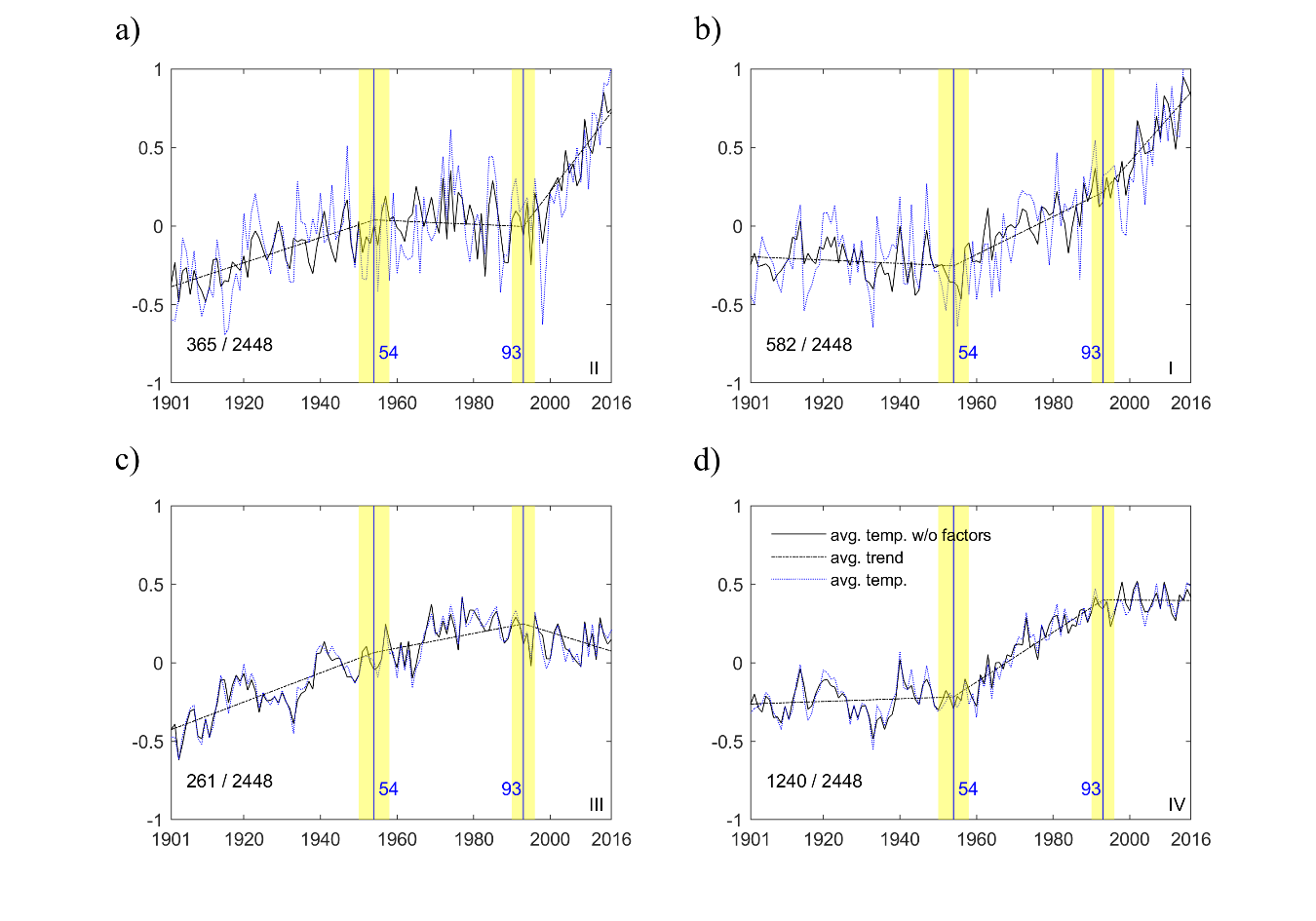


**Figure S5. Average warming trends excluding polar data.** The dash-dot line in each panel shows the average trend for each of cases I ~ IV in Figure 1. Panels a), b), c) and d) denote the cases II, I, III and IV, respectively. The dotted line is the average of annual temperatures excluding polar data and the solid line is the average of annual temperatures excluding polar data without the common factor part ($f_{t}^{'}\lambda_{i}$). The two vertical lines indicate the estimated break dates with the shaded interval being the asymptotic 95% confidence intervals. The fraction in the bottom left corner of each panel shows the number of series for each case.


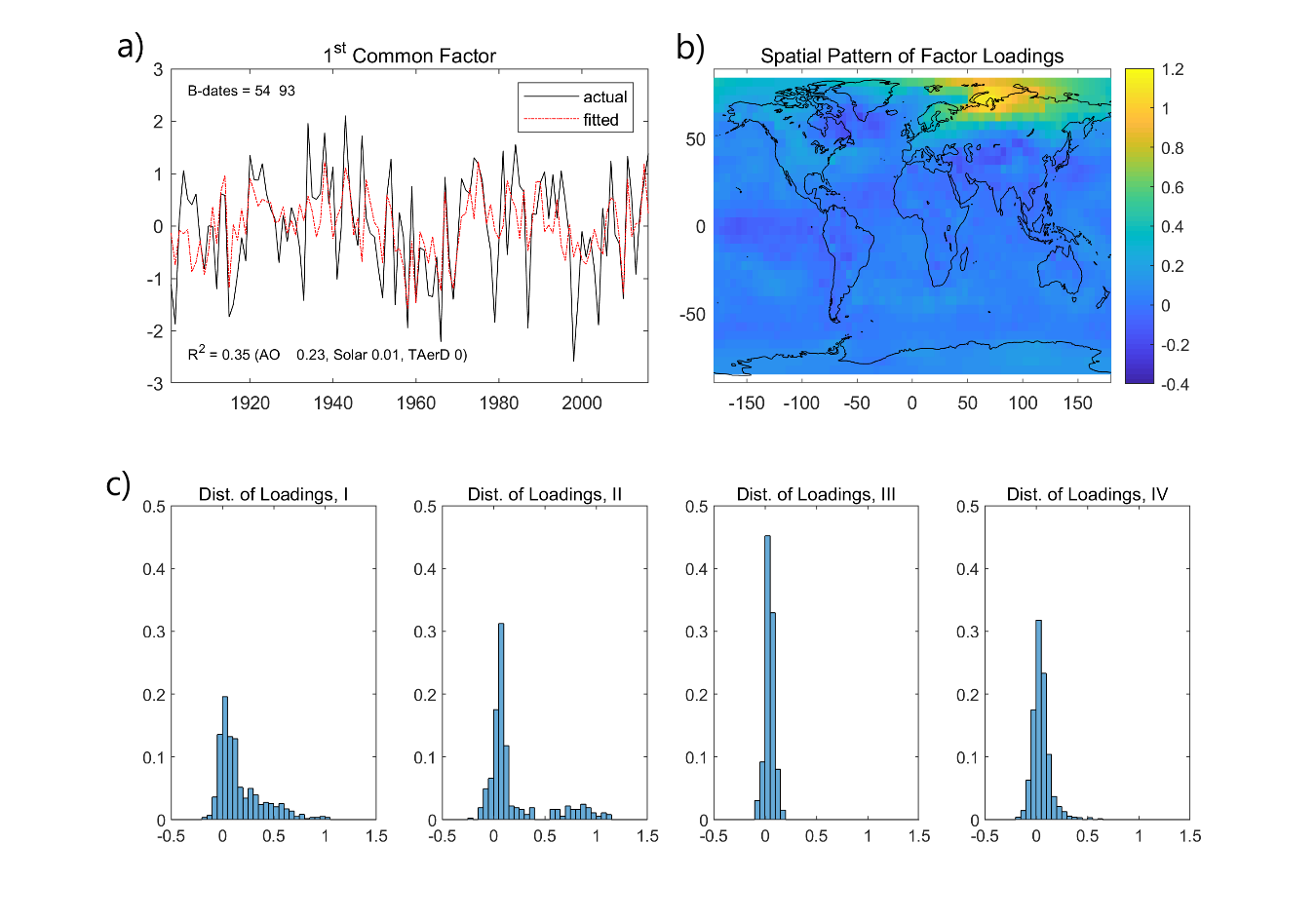


**Figure S6. Time series and spatial pattern of the common factor with annual temperatures and excluding polar data.** The solid black line in panel a) plots the common factor and the red dash-dot line is the fitted line from a regression of the common factor on a constant, AO and forcing factors (see methods). The $R^{2}$ is 0.35 and the numbers in parenthesis are the marginal $R^{2}$ values of the three most significant regressors. Panel b) displays the values of the factor loadings in a color map. Panel c) shows the distribution of the loading values for each of the four cases, I ~ IV in Figure S4. This figure was created using MATLAB R2018a (https://www.mathworks.com/).

**S8. Decomposition of temperature change for each quadrant.**

Using the panel model described in Section S1, the temperature change during the period of the hiatus can be computed and the contribution of each component be estimated. From the panel model $y_{\mathrm{it}}=d_{\mathrm{it}}+f_{t}^{'}\lambda_{i}+e_{\mathrm{it}}$, the decomposition of temperature change for each region is given by:

$\Delta y_{i}=y_{i2016}-y_{i1994}$ (Total temperature change)

$\Delta d_{i}=d_{i2016}-d_{i1994}$ (contribution of the trend component)

$\Delta(f'\lambda_{i}){=f}_{2016}^{'}\lambda_{i}-f_{1994}^{'}\lambda_{i}$ (contribution of the stochastic common factor)

$\Delta e_{i}=e_{i2016}-e_{i1994}$ (contribution of the idiosyncratic error in each region)

Table S1 shows the average values for $\Delta y_{i}$, $\Delta d_{i}$, $\Delta\left( f\lambda_{i} \right)$, and $\Delta e_{i}$ for each quadrant. The largest temperature change occurs in quadrants I and II and the contribution of the trend component is dominant (about 80% of total temperature change), followed by the contribution of the common factor. For locations in quadrant IV a hiatus in the warming, is present, i.e., an almost null contribution from the trend component, consistent with what has been previously reported in the literature^3,4^. The dominant contribution to the slight warming in locations in this quadrant comes from the common factor component and is only 0.05ºC. The locations in quadrant IV illustrate the WACCE pattern in conjunction with the results in quadrants I and II. The locations in quadrant III also experienced a hiatus over this period, showing the smallest warming of across quadrants. The locations in quadrants III and IV, where the hiatus in the warming is present, are composed in a large part by oceans, which suggests that some of the hiatus was produced by a redistribution of heath in the oceans^5,6^. The trend component contributes to a slight cooling which is partially compensated by idiosyncratic climate variability and a small contribution from the common factor component.

|  | $\Delta y$ | $\Delta d$ | $\Delta(f^{'}\lambda)$ | $\Delta e$ |
| --- | --- | --- | --- | --- |
| I | 0.850 | 0.704 | 0.244 | -0.098 |
| II | 0.860 | 0.702 | 0.244 | -0.085 |
| III | 0.016 | -0.167 | 0.054 | 0.128 |
| IV | 0.086 | -0.003 | 0.059 | 0.030 |

Table S1. Estimates of the average contribution of the trend component $\Delta d_{i}$, common factor $\Delta\left( f\lambda_{i} \right)$, and idiosyncratic error $\Delta e_{i}$ to total temperature change $\Delta y$, during the 1994-2016 period.

References

1. Kim, D. Estimating a common deterministic time trend break in large panels with cross sectional dependence. *J. Econom.* **164**, 310–330 (2011).

2. Ahn, S. & Horenstein, A. R. Eigenvalue ratio test for the number of factors. *Econometrica* **81**, 1203–1227 (2013).

3. Cohen, J. *et al.* Divergent consensuses on Arctic amplification influence on midlatitude severe winter weather. *Nat. Clim. Chang.* **10**, 20–29 (2020).

4. Francis, J. A. & Vavrus, S. J. Evidence for a wavier jet stream in response to rapid Arctic warming. *Environ. Res. Lett.* **10**, 014005 (2015).

5. Kosaka, Y. & Xie, S. P. Recent global-warming hiatus tied to equatorial Pacific surface cooling. *Nature* **501**, 403–407 (2013).

6. Fyfe, J. C. *et al.* Making sense of the early-2000s warming slowdown. *Nat. Clim. Chang.* **6**, 224–228 (2016).
